# Synthesis, Physicochemical Properties, and Antimicrobial Studies of Iron (III) Complexes of Ciprofloxacin, Cloxacillin, and Amoxicillin

**DOI:** 10.1155/2014/735602

**Published:** 2014-11-19

**Authors:** Fabian I. Eze, Uzoechi Ajali, Pius O. Ukoha

**Affiliations:** ^1^Department of Pharmaceutical and Medicinal Chemistry, University of Nigeria, Nsukka 410001, Nigeria; ^2^Department of Pure and Industrial Chemistry, University of Nigeria, Nsukka 410001, Nigeria

## Abstract

Iron (III) complexes of ciprofloxacin, amoxicillin, and cloxacillin were synthesized and their aqueous solubility profiles, relative stabilities, and antimicrobial properties were evaluated. The complexes showed improved aqueous solubility when compared to the corresponding ligands. Relative thermal and acid stabilities were determined spectrophotometrically and the results showed that the complexes have enhanced thermal and acid stabilities when compared to the pure ligands. Antimicrobial studies showed that the complexes have decreased activities against most of the tested microorganisms. Ciprofloxacin complex, however, showed almost the same activity as the corresponding ligand. Job's method of continuous variation suggested 1 : 2 metals to ligand stoichiometry for ciprofloxacin complex but 1 : 1 for cloxacillin complex.

## 1. Introduction

Many drugs possess modified pharmacological, toxicological, and physicochemical properties when administered in the form of metal complexes [1]. Physicochemical properties of drugs are very pertinent to dosage forms and drug delivery and complex formation affects these properties, sometimes to advantage and sometimes adversely. Among the properties that may be altered upon complex formation are solubility, energy absorption, stability, partitioning behaviour, and chemical reactivity [2–4]. For many systems, it has been shown that the complex provides faster dissolution and greater bioavailability than the physical mixture. The processing characteristics (physical state, stability, flow ability, etc.) of the complexes may also be better than those of the free drugs [5]. In some cases, complexation has been found to improve biological activity [6–11].

Quinolones are complexing agents for a variety of metal ions including alkaline earth and transition metal ions. Reports indicate that the coordination of quinolones to metal ions such as Cu (II), Mg (II), and Ca (II) appears to be important for the activity of the quinolone antibiotics [11–13]. Coordination compounds may also release valuable trace elements needed for maintenance of life when they are administered as drugs.

The structure-activity relationship of drugs could be predicted by complexation. If a particular biological activity of a drug is lost or diminished on complexation with metal ions, it would be reasonable to suggest that one or more of the groups bonded to the metal is necessary for the activity. Drug complexation experiments can also help medicinal chemists to predict some dosage form incompatibilities, explain the mode of action of some drugs, and devise new methods of drug analysis.

## 2. Materials and Methods

### 2.1. Chemicals and Equipment

All chemicals and solvents used were AnalaR grade. All melting points were taken on a melting point apparatus (Electrothermal, England). Magnetic stirrer (Gallenkamp, England), UV-visible spectrometer (Jenway 6305, Barlowood Sci. Ltd., Dunmow), pH meter (Jenway, Dunmou), and electronic weighing balance (Adventurer, OHAUS Corp., China) were used.

### 2.2. Microorganisms

Clinical isolates of the following microorganisms:* Staphylococcus aureus, Pseudomonas aeruginosa, Bacillus subtilis, Escherichia coli, Salmonella typhi, Shigella *spp*., Aspergillus niger,* and* Candida albicans,* were used. They were maintained in the Department of Pharmaceutics, University of Nigeria, Nsukka, Nigeria.

### 2.3. Synthesis of Iron (III) Complex of Ciprofloxacin

Ciprofloxacin HCl (1 mmole) was dissolved in a minimum quantity of distilled water. To this solution FeCl_3_ (0.5 mmole) dissolved in absolute ethanol was added. The mixture was stirred continuously with magnetic stirrer at room temperature for 3 hours. The resulting red solution was transferred into an evaporating dish and allowed to evaporate slowly at room temperature for one week. The red crystals formed were purified by recrystallizing in a minimum quantity of ethanol and weighed.

### 2.4. Synthesis of Iron (III) Complex of Cloxacillin

Cloxacillin powder (1 mmole) was dissolved in dioxane (100 mL). To this solution an ethanolic FeCl_3_ solution containing 162.5 mg of  FeCl_3_ in ethanol (10 mL) was added. The mixture was stirred continuously for 4 hours at room temperature at the end of which clay-brown crystals separated from the solution. The mixture was filtered and the crystals were washed thoroughly with dioxane, dried in a desiccator, and weighed.

### 2.5. Synthesis of Iron (III) Complex of Amoxicillin

Amoxicillin powder (1 mmole) was dissolved in methanol (100 mL) in a beaker. FeCl_3_ (1 mmole) was put in another beaker containing ethanol (10 mL). The two solutions were put in a round bottom flask and stirred with a magnetic stirrer under reflux maintained at 40°C for 4 hours. The resulting green solution, which was foaming, was transferred into an open beaker and allowed to stand for 1 week. The green crystals obtained were washed thoroughly with small quantity of ethanol, dried, and weighed.

### 2.6. Determination of Stoichiometry of the Complexes

Job's method of continuous variation [14, 15] was followed. A mixture of each ligand and ferric chloride solution was stirred for 3 hours and allowed to equilibrate. The absorbances were read at the *λ*-max of each complex.

### 2.7. Determination of Aqueous Solubility

Saturated solution (10 mL) of each of the complexes and ligands at ambient temperature (28°C) was evaporated to dryness in an evaporating dish. The mass of the solid left in each case was determined. The solubilities were calculated using the relation [16]:

(1) $$\begin{array}{c} S=\frac{\text{mass}}{\text{volume}}\times 1000. \end{array}$$

The solubilities of the complexes were compared with those of the ligands.

### 2.8. Thermal and Acid Stabilities

The relative thermal and acid stabilities of the complexes were determined spectrometrically. Dilute solutions (0.1 mg/mL) were prepared, their absorption spectra were generated, and the *λ*-max in each case was noted. The solution of each complex was divided into seven equal portions and the temperature regulated to 20°C, 30°C, 40°C, 50°C, 60°C, 70°C, and 80°C, respectively, for a period of 24 hours. The absorbance in each case was then measured. Similarly, the same concentration of these solutions was prepared at the pH range of   1–6 and the absorbance was measured. These changes in absorbance with temperature and pH, assuming that Beer-Lambert's law is obeyed, are a measure of the stability of the complexes.

### 2.9. Antimicrobial Studies on the Complexes

The ligands and complexes were assayed for antimicrobial activity by the agar well diffusion [17, 18] and dilution methods, respectively [19–21]. The culture media employed were nutrient agar, for bacteria, and Sabouraud's dextrose agar, for fungi. The microorganisms had been maintained by weekly subculturing and incubation. A 24-hour old culture of each test organism was employed. A constant cell population size of 106 CFU/mL was used throughout the studies. The cell size was established by dilution and standardization through comparison with MacFalan optical density.

Solution of the complexes and pure ciprofloxacin HCl were made in distilled water and those of other ligands in DMSO. Each sample was tested at the same concentration level of 5 mg/mL. The plates were incubated at 32°C for 24 hours (for bacteria) and at 25°C for 48 hours (for fungi). Double determination was made for each sample and mean values of clear inhibition zone diameter (IZD) were measured and recorded. Six dilutions of each of the solutions of ligands and complexes were made and the IZD of each was measured. A linear plot of IZD^2^ against logarithm of concentration was obtained for each organism and test sample and the minimum inhibitory concentration (MIC) was determined.

## 3. Results and Discussions

### 3.1. Yield and Physical Properties

The percentage yield and some physical properties of the iron (III) complexes of ciprofloxacin (CPF-Fe^3+^), cloxacillin (Clox-Fe^3+^), and amoxicillin (Amox-Fe^3+^) are given in Tables 1 and 2. The solubilities were determined at 28°C.

### 3.2. Relative Thermal Stability

The absorbance of 0.1 mg/mL solutions of each of the ligands and their iron (III) complexes at different temperatures is given in Tables 3 and 4. These changes in absorbance with temperature are a measure of the thermal stability of the drugs.

### 3.3. Relative Acid Stability

The absorbance of 0.1 mg/mL solution of each of the ligands and their iron (III) complexes at different pHs is given in Tables 5 and 6. The changes in absorbance with pH are a measure of the acid stability of the compounds.

Complexation offers a useful means of manipulation of the redox potentials of drug molecules, which determines reactivity and, hence, the stability of the molecules [4]. The higher the redox potential of a substance, the more the reactivity and, hence, lower stability and vice versa. From the results of absorbance of the complexes and ligands at different temperatures and pH, the differences in absorbance were very significant in the ligands but not very significant in the complexes even at very drastic conditions (80°C and pH 1). This shows that the concentration of these complexes was still high at such adverse conditions. This may be as a result of the greater ability of the complexes to withstand acid medium (less ease of acid hydrolysis). Pure ciprofloxacin and penicillins are not appreciably stable in aqueous and acid media. Therefore, we can conclude that these complexes are more stable than the pure ligands and hence have advantage over the ligands as drugs.

### 3.4. Results of Antimicrobial Studies

The inhibition zone diameter (IZD) and minimum inhibitory concentration (MIC) of the ligands and complexes are given in Tables 7 and 8, respectively.

Ciprofloxacin complex shows improved antibacterial activity against* Staphylococcus aureus* and* Bacillus subtilis* when compared to that of the ligand. It had almost the same activity against* Pseudomonas aeruginosa*,* Escherichia coli,* and* Shigella *spp. as the ligand and lower activity against* Salmonella typhi* when compared to that of the ligand as shown in the MIC result. Iron (III) complexes of cloxacillin and amoxicillin showed decreased antibacterial and antifungal activities when compared to those of the corresponding ligands. This could be attributed to loss of some essential pharmacophoric moieties due to coordination with the metal ion. The sites used for dative bonding with the central metal ion are no longer available for binding with the biological receptors in the microorganisms. This could also be explained on the basis of action of some antibiotics. Several antibiotics have the property of forming chelates with metals ions needed for normal functioning of enzyme system in the microorganisms rendering them inactive. The complexed form of the drug is not capable of such chelation since the chelation sites are already occupied. A deviation from the optimal lipophilicity due to increased aqueous solubility can also account for the activity.

### 3.5. Results of Stoichiometry by Job's Method of Continuous Variation

**Figure 1:**
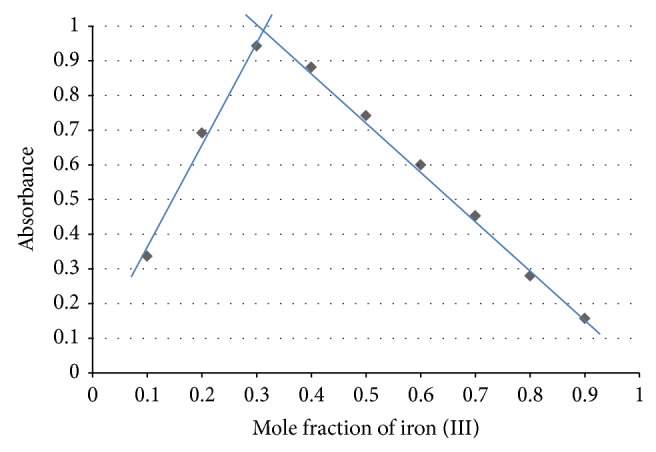
Job's plot for ciprofloxacin complex. The mole fraction of iron (III) at the intersection of the two straight lines is 0.32. The mole ratio is 3.2 : 6.8 which corresponds to 1 : 2 metal-ligand stoichiometry.

**Figure 2:**
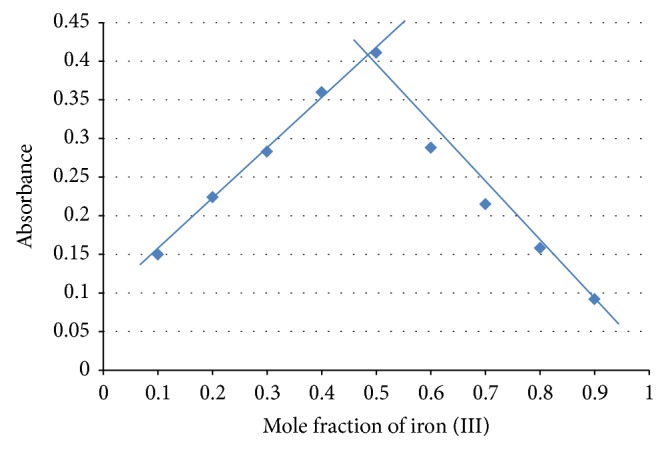
Job's plot for cloxacillin complex. The mole fraction of iron (III) at the intersection of the two straight lines is 0.48. The mole ratio is 4.8 : 5.2 which corresponds to 1 : 1 metal-ligand stoichiometry.

Job's plot for ciprofloxacin and cloxacillin is given in Figures 1 and 2, respectively, while the raw results are given in Tables 9 and 10, respectively. These suggest 1 : 2 and 1 : 1 metal-ligand stoichiometry, respectively.

## 4. Conclusion

Complexation improves the aqueous solubility and thermal and acid stabilities of ciprofloxacin, cloxacillin, and amoxicillin. The improvement in solubility can be applied in parenteral administration of these drugs. Loss of these drugs to deterioration during storage may be minimized if they are stored in the form of their coordination complexes. Complexes of these drugs, therefore, have advantage over the pure drugs due to this inherent stability. Oral administration of these drugs in their complex forms may reduce absorption in the lipid layers. This is because the increased aqueous solubility would result in lower partition coefficient. Coadministration of these drugs with iron supplements is therefore discouraged because complexation may occur* in vivo*.

## Figures and Tables

**Table 1:** Yield and physical properties of the iron (III) complexes.

	Percentage yield (%)	Melting point (°C)	*λ*-max (nm)	Metal-ligand stoichiometry	Estimated molecular weight (g mol^−1^)
CPF-Fe^3+^	80	172	443	1 : 2	891
Clox-Fe^3+^	95	150	385	1 : 1	586
Amox-Fe^3+^	63.5	142	440	—	—

**Table 2:** Aqueous solubility.

Compound	Solubility (gdm^−3^)	
	Pure ligand	Fe^3+^ complex
Ciprofloxacin	≪1	>5
Cloxacillin	Negligible	1.8
Amoxicillin	Negligible	2.0

**Table 3:** Absorbance of the complexes at different temperatures.

Temperature	20°C	30°C	40°C	50°C	60°C	70°C	80°C	*λ*-max (nm)
Complex								
CPF-Fe^3+^	0.609	0.686	0.689	0.686	0.665	0.635	0.629	443
Clox-Fe^3+^	0.114	0.122	0.122	0.120	0.109	0.102	0.083	385
Amox-Fe^3+^	0.095	0.109	0.128	0.133	0.126	0.122	0.105	440

**Table 4:** Absorbance of the pure ligands at different temperatures.

Temperature	20°C	30°C	40°C	50°C	60°C	70°C	80°C	*λ*-max (nm)
Ligand								
Ciprofloxacin	0.348	0.362	0.311	0.240	0.170	0.104	0.081	315
Cloxacillin	0.721	0.727	0.683	0.455	0.337	0.190	0.184	290
Amoxicillin	0.661	0.664	0.643	0.428	0.369	0.312	0.126	254

**Table 5:** Absorbance of the complexes at different pHs.

Complex	pH 1	pH 2	pH 3	pH 4	pH 5	pH 6	pH 7	*λ*-max (nm)
CPF-Fe^3+^	0.216	0.246	0.256	0.291	0.360	0.362	0.368	443
Clox-Fe^3+^	0.008	0.013	0.053	0.055	0.072	0.110	0.122	385
Amox-Fe^3+^	0.645	0.511	0.452	0.360	0.124	0.118	0.109	440

**Table 6:** Absorbance of the ligands at different pHs.

Ligand	pH 1	pH 2	pH 3	pH 4	pH 5	pH 6	pH 7	*λ*-max (nm)
Ciprofloxacin	0.150	0.152	0.171	0.220	0.300	0.315	0.360	315
Cloxacillin	0.437	0.449	0.550	0.554	0.696	0.704	0.722	290
Amoxicillin	0.387	0.404	0.499	0.511	0.603	0.620	0.664	254

**Table 7:** Inhibition zone diameter (in mm).

	Ciprofloxacin		Cloxacillin		Amoxicillin	
	Ligand	CPF-Fe^3+^	Ligand	Clox-Fe^3+^	Ligand	Amox-Fe^3+^
*S. aureus *	44.0	48.0	31.5	—	30.0	—
*B. subtilis *	45.0	46.0	32.0	—	30.0	—
*P. aeruginosa *	48.5	48.0	20.0	—	28.0	—
*E. coli *	40.0	45.0	28.0	—	33.0	—
*S. typhi *	48.5	11.0	27.0	—	35.5	—
*Shigella* spp.	30.0	27.0	30.0	—	30.0	—
*A. niger *	—		30.0	—	35.0	—
*C. albicans *	—		—	—	—	—

**Table 8:** Minimum inhibitory concentration (MIC) in *μ*g/mL.

	Ciprofloxacin		Cloxacillin		Amoxicillin	
	Ligand	CPF-Fe^3+^	Ligand	Clox-Fe^3+^	Ligand	Amox-Fe^3+^
*S. aureus *	12.0	7.9	12.5	—	12.5	—
*B. subtilis *	6.3	5.0	12.5	—	12.5	—
*P. aeruginosa *	3.2	3.2	22.0	—	14.0	—
*E. coli *	2.8	2.8	14.5	—	12.5	—
*S. typhi *	0.18	177	14.5	—	11.0	—
*Shigella* spp.	14	12.5	12.5	—	11.0	—
*A. niger *	—	—	14.5	—	15.0	—
*C. albicans *	—	—	—	—	—	—

**Table 9:** Absorbance of ciprofloxacin complex at different mole fractions of iron (III).

Metal-ligand ratio	Mole fraction of Fe^3+^	Absorbance
1 : 9	0.1	0.337
2 : 8	0.2	0.692
3 : 7	0.3	0.943
4 : 6	0.4	0.882
5 : 5	0.5	0.742
6 : 4	0.6	0.600
7 : 3	0.7	0.453
8 : 2	0.8	0.280
9 : 1	0.9	0.157

**Table 10:** Absorbance of cloxacillin complex at different mole fractions of iron (III).

Metal-ligand ratio	Mole fraction of Fe^3+^	Absorbance
1 : 9	0.1	0.150
2 : 8	0.2	0.224
3 : 7	0.3	0.283
4 : 6	0.4	0.360
5 : 5	0.5	0.411
6 : 4	0.6	0.288
7 : 3	0.7	0.215
8 : 2	0.8	0.158
9 : 1	0.9	0.092
